# *Candida albicans SUR7 *contributes to secretion, biofilm formation, and macrophage killing

**DOI:** 10.1186/1471-2180-10-133

**Published:** 2010-04-30

**Authors:** Stella M Bernardo, Samuel A Lee

**Affiliations:** 1 Section of Infectious Diseases, New Mexico Veterans Healthcare System, Albuquerque, NM, USA; 2 Division of Infectious Diseases, Department of Medicine, University of New Mexico Health Science Center, Albuquerque, NM, USA

## Abstract

**Background:**

*Candida albicans SUR7 *has been shown to be required for plasma membrane organization and cell wall synthesis, but its role in virulence is not known. Using a bioinformatics strategy, we previously identified several novel putative secretion pathway proteins potentially involved in virulence, including the *C. albicans *homolog of the *Saccharomyces cerevisiae *endocytosis-related protein Sur7p. We therefore generated a *C. albicans sur7*Δ null mutant and examined its contribution to key virulence attributes.

**Results:**

Structurally, the *C. albicans sur7*Δ mutant was impaired in response to filamentation-inducing conditions, and formed aberrant hyphae with extensive accumulation of plasma membrane-derived structures within the cell. Absence of *SUR7 *resulted in a temperature-sensitive growth defect at high temperatures (42°C), which was partially rescued by addition of NaCl. We next examined the role of the *SUR7 *paralog *C. albicans FMP45 *in this temperature-sensitive phenotype. Analysis of *C. albicans *Fmp45p-GFP demonstrated co-localization of Fmp45p with Sur7p and increased fluorescence in the plasma membrane in the presence of high salt. We next focused on key virulence-related phenotypes. The *C. albicans sur7*Δ null mutant exhibited secretory defects: reduced lipase secretion, and increased levels of secreted Sap2p. The null mutant was hyper-susceptible to sub-inhibitory concentrations of caspofungin, but not amphotericin B and 5-fluorocytosine. Functionally, the *sur7*Δ mutant demonstrated increased adhesion to polystyrene and of note, was markedly defective in biofilm formation. In an *in vitro *macrophage model of virulence, the *sur7*Δ mutant was impaired in macrophage killing.

**Conclusions:**

Plasma membrane and cell wall organization are important for cell morphology, and alterations of these structures contributed to impairment of several key virulence-associated phenotypes in the *C. albicans sur7*Δ mutant.

## Background

*C. albicans SUR7 *shares 44% identity and 65% similarity with *S. cerevisiae SUR7*. *S. cerevisiae SUR7 *encodes a predicted integral membrane protein with an N-terminal signal sequence and four transmembrane domains, and is a member of a family of proteins that also includes Yn1194p, Ydl222p, and Ylr414cp [1,2]. Sur7p localizes to large, immobile, stable cortical patches on the plasma membrane, termed "eisosomes" which mark sites of endocytosis [3,4]. Deletion of *S. cerevisiae SUR7 *resulted in a strain with a defect in sporulation and altered plasma membrane sphingolipid content [4].

Alvarez and Konopka [5] identified *C. albicans *Sur7p in a detergent-resistant fraction of the plasma membrane in a proteomics study on *N*-acetylglucosamine-induced proteins. Recently, they generated a *C. albicans sur7*Δ knockout mutant which is characterized by aberrant cell wall organization [2]. Specifically, lack of *SUR7 *in *C. albicans *results in mislocalization of actin and septin, and abnormal cell wall material protruding into and forming structures within the cytoplasm. However, from a phenotypic standpoint, little is known regarding the role of *C. albicans SUR7 *in pathogenesis.

A number of *C. albicans *virulence-related secreted proteins that remain associated with the plasma membrane or cell wall have been identified, including the outer mannoprotein Hwp1p [6], adhesins encoded by the *ALS *family of genes [7], and membrane proteins encoded by the pH-responsive genes *PHR1 *and *PHR2 *[8-11]. However, a genome-wide understanding of *Candida *secretory pathway proteins and virulence is still limited. Previously, we took advantage of SignalP v2.0 [12,13] and a series of additional validated predictive algorithms to define a computational secretome of *C. albicans *from its entire genome [14]. In addition to identifying putative soluble secretory proteins, we also identified a number of putative and known membrane and cell-wall associated proteins [14]. We next compared these databases with published genome-wide expression profiling data to identify candidate virulence-related genes. Fradin et al. [15] performed genomic expression profiling in *C. albicans *exposed *in vitro *to blood and *in vivo *during infection in a standard mouse model of disseminated candidiasis and identified groups of genes highly expressed under these conditions. When compared with the dataset of predicted secretion pathway ORFs, a number of virulence-related genes were concordant, including Hwp1p and the Als family of adhesins [6,7], Phr1p [8], Sap9p [16], Sod5p [17,18], and Sun41p [19-21]. Thus, we identified known soluble secreted and membrane-associated secretion pathway proteins important for virulence, supporting our approach as a method to identify candidate virulence-related genes.

We also identified *orf19.3414*, which is predicted to encode a secretion pathway protein homologous to the *S. cerevisiae *endocytosis-related gene *SUR7 *[1]. As we independently identified *C. albicans SUR7 *in our screen for candidate virulence-related genes, we used a reverse genetic approach to investigate the role of *C. albicans SUR7 *in attributes related to virulence in order to define its role in pathogenesis.

## Results

### The temperature sensitive growth defect of the *Candida albicans sur7*Δ mutant is partially rescued by high salt

**Figure 1:**
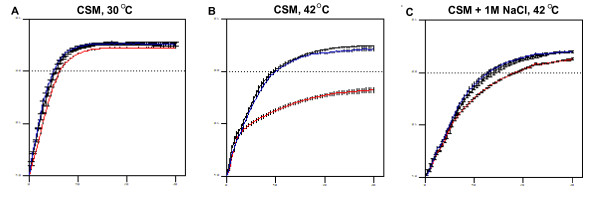
**Characterization of growth of the *sur7*Δ null mutant strain**. The growth of strains DAY185 (black), *sur7*Δ (red), and *sur7*Δ+*SUR7 *(blue) were compared under different conditions. Standard growth conditions at 30°C in complete synthetic medium (CSM) supplemented with uridine is shown in (**A**). Growth at extreme temperature (42°C) was also tested in CSM supplemented with uridine (**B**) and CSM with 1 M NaCl supplemented with uridine (**C**). At high temperatures (42°C), the impaired growth of the *sur7*Δ null mutant strain was statistically significant compared to the control strains. All growth curves were performed in triplicate, with Log_10 _of OD_600 _plotted against Time (hours). Corresponding error bars are indicated. Table 2 lists the calculated doubling time of each strain under each condition presented here.

We generated a *C. albicans sur7*Δ homozygous null mutant by PCR-mediated gene disruption [22,23], SMB3-H, followed by construction of an isogenic complemented strain, SMB3-R (Table 1). Before proceeding with phenotypic characterizations of the *sur7*Δ null mutant, we assessed the growth of each strain by calculating doubling times. Growth curves and the resulting doubling times are presented in Fig. 1 and Table 2, respectively. In rich medium, there was no statistically significant difference (p > 0.05) between the calculated doubling times of the *C. albicans sur7*Δ mutant, prototrophic control strain DAY185, and the isogenic complemented strain (Fig. 1A and Table 2). Growth in response to high osmotic stress (1.0 M NaCl or 2.5 M glycerol) was the same as that of the control strains when incubated at either 30 or 37°C (data not shown). Interestingly, when incubated at 42°C, growth of the *sur7*Δ null mutant strain was markedly impaired, in contrast to the control and *SUR7 *complemented strains (Fig. 1B). The *sur7*Δ null mutant grew at one-third the rate of the wild-type control strains (Table 2). Unexpectedly, the *sur7*Δ null mutant's inability to grow at 42°C was partially rescued when grown under conditions of high salt (1.0 M NaCl; Fig. 1C); differences in doubling time compared to the control strains were statistically significant (p < 0.001).

**Table 1:** *Candida albicans *strains used in this study.

Strain	Parent	Relevant genotype	Source
DAY185		*ura3*Δ::λ*imm434*/*ura3*Δ::λ*imm434 his1*::*hisG*/*HIS1*::*his1*::*hisG arg4*::*hisG*/*ARG4*::*URA3*::*arg4*::*hisG SUR7/SUR7*	[48]

BWP17		*ura3*Δ/*ura3*Δ *arg4*Δ/*arg4*Δ *his*Δ/*his1*Δ *SUR7/SUR7 FMP45/FM45*	[22]

SMB2	BWP17	*ura3*Δ/*ura3*Δ *arg4*Δ/*arg4*Δ *his*Δ/*his1*Δ *SUR7/sur7*Δ::*dpl200-URA3-dpl200*	This study

SMB3	SMB2	*ura3*Δ/*ura3*Δ *arg4*Δ/*arg4*Δ *his*Δ/*his1*Δ *sur7*Δ::*dpl200-URA3-dpl200/sur7*Δ::*ARG4*	This study

SMB3-H	SMB3	*ura3*Δ/*ura3*Δ *arg4*Δ/*arg4*Δ *his*Δ/*his1*Δ *HIS1* *sur7*Δ::*dpl200-URA3-dpl200/sur7*Δ:: *ARG4*	This study

SMB3-R	SMB3	*ura3*Δ/*ura3*Δ *arg4*Δ/*arg4*Δ *his*Δ/*his1*Δ*HIS1*::*SUR7* *sur7*Δ::*dpl200-URA3-dpl200/sur7*Δ:: *ARG4*	This study

B-FMP45gfp	BWP17	*ura3*Δ/*ura3*Δ *arg4*Δ/*arg4*Δ *his*Δ/*his1*Δ *SUR7/SUR7 FMP45/FM45-GFP-HIS1*	This study

sΔ-FMP45gfp	SMB3	*ura3*Δ/*ura3*Δ *arg4*Δ/*arg4*Δ *his*Δ/*his1*Δ *FMP45/FM45-GFP-HIS1 sur7*Δ::*dpl200-URA3-dpl200/sur7*Δ:: *ARG4*	This study

*SUR7*-YFP	BWP17	*ura3*Δ/*ura3*Δ *arg4*Δ/*arg4*Δ *his*Δ/*his1*Δ *FMP45/FM45 SUR7/SUR7-YFP-HIS1*	This study

*SUR7*-GFP	BWP17	*ura3*Δ/*ura3*Δ *arg4*Δ/*arg4*Δ *his*Δ/*his1*Δ *FMP45/FM45 SUR7/SUR7-GFP-HIS1*	This study

*SUR7*-YFP *FMP45*-GFP	SUR7-YFP	*ura3*Δ/*ura3*Δ *arg4*Δ/*arg4*Δ *his*Δ/*his1*Δ *SUR7/SUR7-YFP-HIS1 FMP45/FM45-GFP-URA3*	This study

**Table 2:** Calculated doubling times of *C. albicans *strains under different conditions of growth.

	Doubling time of each strain (hours)		
**Growth conditions**	**Wild-type** **(DAY185)**	***sur7*Δ** **(SMB3-H)**	***sur7*Δ + *SUR7*** **(SMB3-R)**

30°C, CSM (p > 0.05)	2.244 ± 0.070	2.199 ± 0.016	2.168 ± 0.034

42°C, CSM (*p < 0.0001)	3.645 ± 0.066	11.08 ± 0.122 *	3.560 ± 0.055

42°C, CSM + 1 M NaCl (*p < 0.001)	3.145 ± 0.119	3.374 ± 0.072 *	3.000 ± 0.036

* indicates statistical significance of *sur7*Δ compared to wild-type and *SUR7 *complemented strains.

### *C. albicans *Fmp45p-GFP fluorescence intensity increases in the presence of high salt and temperature

**Figure 2:**
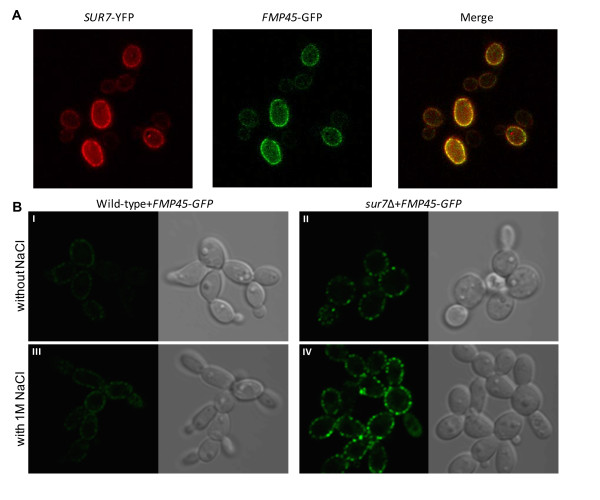
**Induction and cellular localization of Fmp45p-GFP**. (**A**) Spectral cube (fluorescence) images were acquired using the Nuance™ Multispectral Imaging System (CRi) to assess cellular localization of Fmp45p-GFP and Sur7p-YFP in the multi-labelled strain *SUR7*-YFP *FMP45*-GFP. Individual localization is shown for each protein of interest (Sur7p-YFP and Fmp45p-GFP). Sur7p-YFP was artificially rendered in red so that co-localized proteins can be readily distinguished (yellow) in the merged image. (**B**) Localization of Fmp45p-GFP in either the wild-type (BWP17) or *sur7*Δ null (SMB3) background was visualized by laser scanning confocal microscopy. Strains were grown at 42°C at a starting OD_600 _of 0.1 in complete synthetic medium, supplemented with 1.0 M NaCl where required. After 24 h growth, confocal fluorescence images were documented using parameters optimized for imaging the *sur7*Δ *FMP45*-*GFP *strain (sΔ-FMP45gfp) grown in the presence of high salt. Panels show fluorescence and DIC images of strains B-FMP45gfp (I and III, excluding and including salt, respectively) and sΔ-FMP45gfp (II and IV, excluding and including salt, respectively).

In *S. cerevisiae*, transcript levels of the *SUR7 *paralog *YNL194 *is increased in the presence of high salt [24]. Expression of the Ynl194p-GFP fusion protein under its native promoter shifted from barely detectable fluorescence levels to highly detectable levels with the addition of high salt, and the gene product was found to co-localize with *S. cerevisiae *Sur7p [4]. There is only one closely related paralog of *Ca *Sur7p in *C. albicans*, *Ca *Fmp45p (*orf19.6489*), which shares 31% identity and 45% similarity. According to predictions using TMHMM http://www.cbs.dtu.dk/services/TMHMM/, Fmp45p has 4 transmembrane domains similar to Sur7p defined by amino acids 7-29; 106-128; 135-157; 186-208, and 7-29; 104-126; 139-161; 183-205, respectively. These results are similar to data presented by Alvarez et al. [2] who also noted the presence of a conserved Cys-containing motif in *C. albicans *Fmp45p similar to the consensus sequence that is characteristic of members of the claudin family of proteins. To explore the functional relation between *C. albicans SUR7 *and *FMP45*, we created a double-fluorescent labelled strain, *SUR7*-*YFP FMP45*-*GFP*, whose expression of both fusion proteins remain under the control of their native promoters. While the fluorescence emission overlap of YFP and GFP makes it impossible to separate them using conventional epifluorescence imaging, the Nuance™ Multispectral Imaging System (CRi) can distinguish the spectra of the YFP- and GFP-tagged proteins, and produce separate images of Sur7p-YFP and Fmp45p-GFP from the single *SUR7*-YFP *FMP45*-GFP strain. The merged fluorescence images indicate that Fmp45p co-localizes in a punctate pattern with the plasma membrane-bound protein Sur7p (Fig. 2A). These results are similar to that observed in *S. cerevisiae *[4]. We thus hypothesized that under these specific growth conditions (high temperature and salt), the *C. albicans *paralog *FMP45 *may be contributing to a compensatory response to high salt.

To test this hypothesis, we created strains B-FMP45gfp and sΔ-FMP45gfp expressing the Fmp45p-GFP fusion protein in both wild-type and *sur7*Δ null backgrounds, respectively (Table 1). In the wild-type background, Fmp45p-GFP fluorescence intensity is very low, and appears to display a punctate pattern of plasma membrane localization (Fig. 2B, panel I). In the presence of high salt, Fmp45p fluorescence intensity in the *SUR7*^+ ^background is increased (Fig. 2B, panel III). In the *sur7*Δ null background, the fluorescence pattern of Fmp45p-GFP is clearly punctate, similar to the localization of Sur7p, and in the absence of salt an increase in Fmp45p-GFP fluorescence is readily observed (Fig. 2B, panel II). In the presence of high salt (1.0 M NaCl), Fmp45p-GFP fluorescence greatly increased in the *sur7*Δ background and maintained the punctate pattern that is typical of Sur7p localization (Fig. 2B, panel IV). Using Image J software analysis, we quantified the relative fluorescence intensity of all major points around a given cell. The median intensity of each cell with a wild-type (without and with salt) and *sur7*Δ null (without and with salt) background was 212, 279, 491, and 1040, respectively. These measurements are in agreement with visual observation of the images obtained (Fig. 2B). The co-localization of Fmp45p and Sur7p and the increase in fluorescence intensity of Fmp45p-GFP in the presence of 1 M NaCl together suggest that Fmp45p may play a role in tolerance of high salt in the absence of *C. albicans *Sur7p.

### The *Candida albicans sur7*Δ mutant is defective in tolerance to cell wall stress and antifungal agents targeting cell wall components

**Figure 3:**
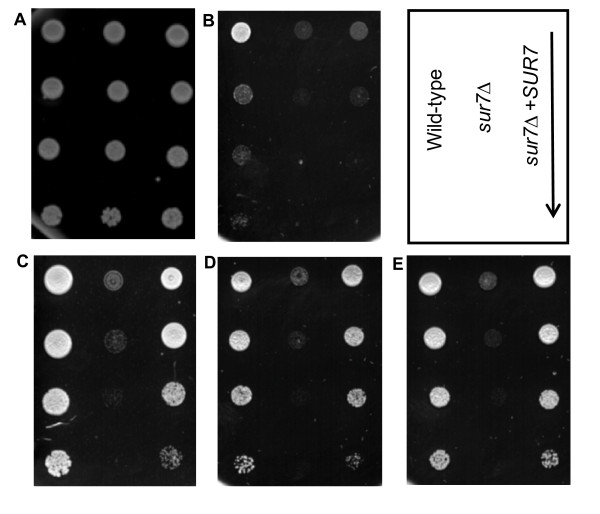
**Cell wall defects of the *sur7*Δ null mutant**. Serial dilutions of overnight cultures were spotted onto different agar media and incubated for 2 days at 30°C. Strains are indicated in the top right diagram with an arrow signifying decreasing cell densities (1 × 10^7^, 2 × 10^6^, 4 × 10^5^, 8 × 10^4 ^and cells ml^-1^) of the strains spotted onto each plate. Normal growth on YPD medium is shown in (**A**). YPD medium containing cell wall perturbing agents such as (**B**) 0.1 μg ml^-1 ^caspofungin, (**C**) 0.02% SDS, (**D**) 200 μg/ml Congo Red, and (**E**) 50 μg ml^-1 ^Calcofluor White are shown.

Next we tested growth in the presence of sub-inhibitory concentrations of several different classes of antifungal agents at 30 and 37°C. No difference was seen in growth in the presence of amphotericin B or 5-fluorocytosine (data not shown). However, the *C. albicans sur7*Δ mutant was more susceptible to sub-inhibitory concentrations of caspofungin (CAS at 0.25 μg/ml; data not shown). We further investigated cell wall integrity in the *sur7*Δ null mutant using a number of cell wall perturbing agents. Serial dilutions of each strain were spotted onto YPD medium containing various concentrations of CAS, SDS, Congo Red, and Calcofluor White. In the absence of *SUR7 *the organism was highly sensitive to each compound tested (Fig. 3). Furthermore, a modest gene dosage effect was suggested, as the degree of sensitivity of the *SUR7*-complemented strain was intermediate between that of the wild-type and *sur7*Δ strains. When tested on the same media, the heterozygous mutant strain (SMB2) exhibited the same degree of sensitivity to cell wall perturbing agents as the *SUR7 *complemented strain (data not shown).

Taken together, these initial studies on the *sur7*Δ mutant indicate an overall defect in cell wall structure, and consequent defects in the ability of the *sur7*Δ mutant to tolerate specific stresses related to cell wall function. We next focused on studying the implications of this defective structure on factors that contribute to virulence, including adhesion, filamentation, protease secretion, and biofilm formation.

### The *C. albicans sur7*Δ mutant has an abnormal response to induction of filamentation and hyphal cells are markedly defective in plasma membrane structure

**Figure 4:**
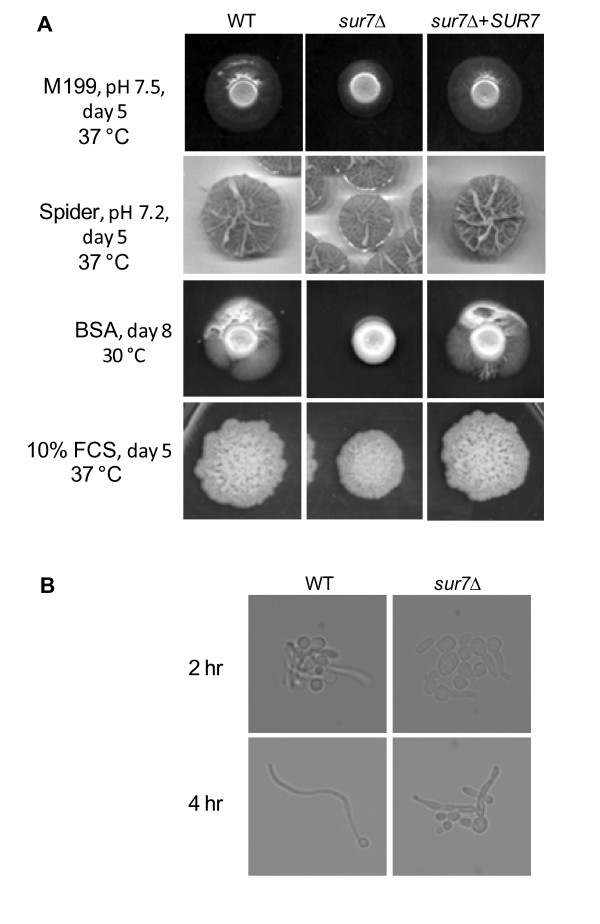
**Filamentation assays on various media**. (**A**) Overnight cultures were spotted onto weak-inducing media such as M199 agar plates, Spider agar, and BSA plates, and monitored daily. Overnight cultures were also spotted onto YPD containing 10% (v/v) fetal calf serum (FCS), a strong inducer of filamentation. Representative figures at the indicated times and incubation temperatures are shown. (**B**) Filamentation was also assayed in liquid media. Inoculums of 5 × 10^6 ^cells ml^-1 ^were incubated at 37°C with constant shaking at 200 rpm. The time of germination, extent of elongation, and overall hyphal morphology were observed and compared between each strain at given time points using standard light microscopy. Results from growth in weak-inducing medium (Spider medium) are shown here at 2 and 4 hours where aberrant branching is evident at the latter timepoint. Standard light microscopy was performed using a 60× and 40× objective for the 2 and 4 hour timepoint, respectively.

An important virulence attribute in *C. albicans *is the ability to switch between yeast, pseudohyphal, and filamentous forms [25-27]. When spotted onto M199 agar, hyphal structures were formed from each colony (Fig. 4A). However, the extent of filamentation was reduced in the *sur7*Δ null mutant compared to DAY185 and the *SUR7 *complemented strain. Similar results were observed when grown on Spider agar medium at 37°C (Fig. 4A). When BSA agar plates were incubated for an extended period of time, filamentous structures emerged from the edge of each colony except in the *sur7*Δ null mutant (Fig. 4A). This reduced filamentation in response to inducing conditions was also seen on solid media containing fetal calf serum (Fig. 4A). In liquid media (YPD supplemented with 10% FCS, high glucose D-MEM with 10% FCS, or RPMI-1640), time of germination and the extent of filament elongation of the *C. albicans sur7*Δ mutant were grossly similar to the wild-type and *SUR7 *complemented strains (data not shown). However, when grown in weak hyphal-inducing liquid Spider medium, a population of yeast cells and hyphae with aberrant morphology and branching was observed (Fig. 4B).

**Figure 5:**
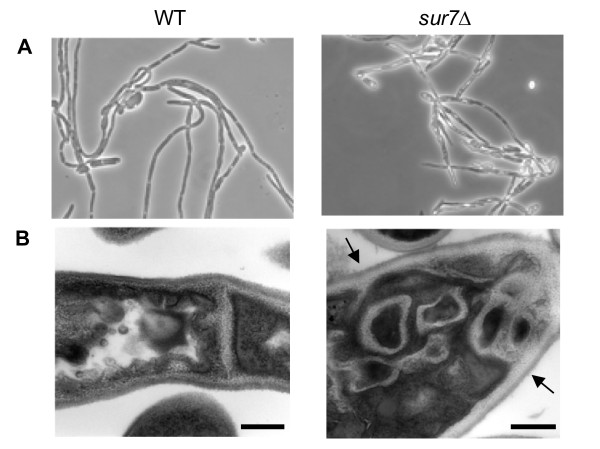
**Cellular morphology of the hyphal form of the *C. albicans sur7*Δ null mutant strain**. (**A**) Filamentation was assessed in RPMI-1640 medium. Medium was inoculated with 5 × 10^6 ^cells ml^-1 ^and incubated at 37°C for 24 h with constant shaking at 200 rpm. Standard light microscopy with a 40× objective was used to visualize the morphology of the filaments formed by wild-type (WT) and *sur7*Δ homozygous null mutant (*sur7*Δ) strains. (**B**) Thin-section electron micrographs of wild-type and *sur7*Δ hyphal cells are shown with arrows indicating abnormal structures similar to that seen in the yeast form of the *sur7*Δ null mutant (Fig. 7B). A size bar is shown to indicate 500 nm.

Next, structures of the filamentous form were compared using light microscopy. After 24 hours of growth, the wild-type (DAY185; Table 1) and *SUR7 *complemented strains produced mature, elongated hyphal cells with clear septa, whereas the *sur7*Δ null mutant produced irregularly shaped hyphae with obvious intracellular invaginations (Fig. 5A). Thin-section electron microscopy demonstrated subcellular structures in the filaments formed by the *sur7*Δ null mutant strain (Fig. 5B) which, as in the yeast form cells (presented in the next section), are likely related to the plasma membrane based on subcellular appearance.

### *C. albicans sur7*Δ mutant hyphal cells are defective in endocytosis

**Figure 6:**
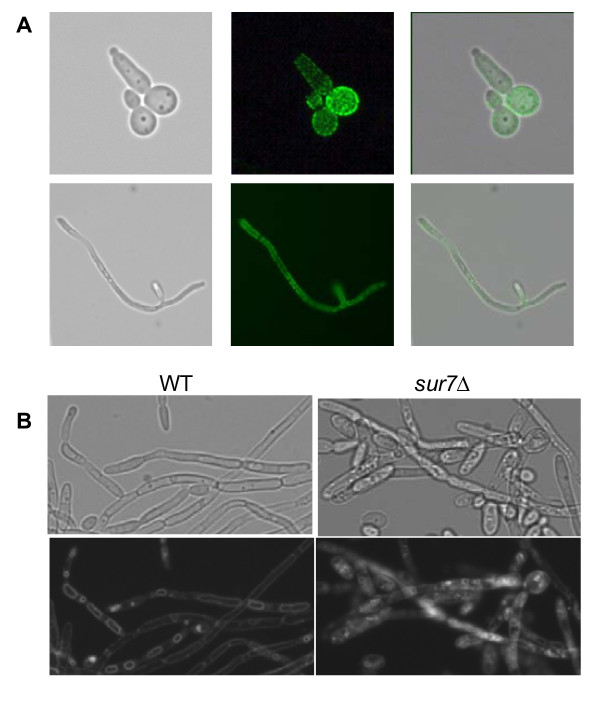
**The role of *SUR7 *in endocytosis in *C. albicans *hyphal form**. (**A**) Fluorescence microscopy was used to assess cellular localization of *C. albicans *Sur7p in the filamentous form of the *SUR7*-GFP strain. Hyphal growth was induced in RPMI-1640 medium at 37°C and protein localization was visualized at stages of early germination (top panel) and mature hyphae formation (bottom panel). Brightfield, green fluorescent, and merged images are shown. Sur7p-GFP is observed at the plasma membrane of the germinating tube, as is the case in yeast cells, but is absent from the growing hyphal tip. (**B**) FM4-64 was used to stain the vacuoles in *C. albicans *hyphae following standard protocols for vacuolar staining of the yeast cells [41].

*S. cerevisiae *Sur7p is a component of eisosomes which mark sites of endocytosis in the plasma membrane [3]. Sur7p is localized to the plasma membrane in the filamentous form of *C. albicans *in a punctate pattern (Fig. 6A), similar to that observed in the yeast form, suggesting retention of its endocytic role in hyphae. Thus, to examine the role of the *C. albicans *Sur7p in endocytosis in filamentous cells, we used the lipophilic membrane dye FM4-64 and visualized its fate using fluorescence microscopy. Since FM4-64 initially binds to the plasma membrane, followed by active endocytosis, the sub-cellular structures stained with FM4-64 in the *sur7*Δ mutant (Fig. 6B) appear to correspond to the aberrant structures accumulating in filaments seen on electron microscopy (Fig. 5B).

**Figure 7:**
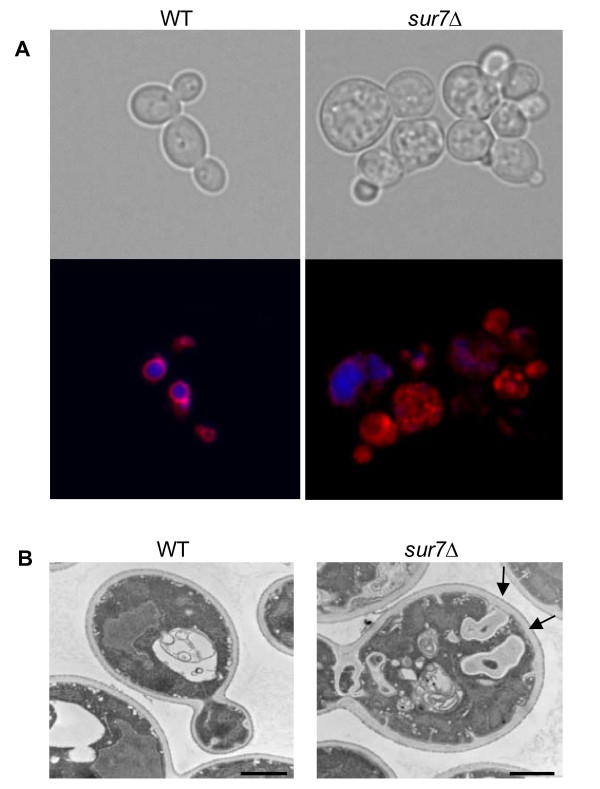
**Vacuolar structure in the *C. albicans sur7*Δ mutant**. (**A**) Carboxy-DCFDA was used in addition to FM4-64 to differentiate between vacuolar and non-vacuolar structures stained by FM4-64 in *C. albicans *yeast cells. Upon active endocytosis, FM4-64 stains the vacuolar membrane whereas CDCFDA is passively diffused into the cell and into the vacuolar lumen. Images were taken following sufficient incubation that allowed each dye to reach the vacuole. (**B**) Thin-section electron micrographs of yeast cells of the wild-type and *sur7*Δ null mutant strains are shown with arrows indicating abnormal invagination of the plasma membrane and subcellular structures of plasma membrane origin. A size bar is shown to indicate 1 μm.

In order to further define the origin of these aberrant structures, we stained these cells in the yeast form with the vacuolar luminal dye carboxy-DCFDA (CDCFDA) (Fig. 7A). CDCFDA reaches the vacuole via passive diffusion in contrast to FM4-64 which is internalized through the endocytic pathway. CDCFDA and FM4-64 stained the vacuolar lumen and membrane, respectively, in control strains, DAY185 and the *SUR7 *complemented strain. In contrast, most of the FM4-64 dye did not reach the vacuolar membrane of the *sur7*Δ null mutant, but instead remained in non-vacuolar structures as evidenced by the lack of co-staining with CDCFDA (Fig. 7A). The vacuole of the *sur7*Δ null mutant is intact as shown by CDCFDA staining, and by thin section electron microscopy (Fig. 7B). Taken together these results clearly demonstrate that the structures stained by FM4-64 are not fragmented vacuoles, and are plasma membrane-derived.

Thus, from a structural perspective, the overall plasma membrane architecture of both yeast and hyphal cells, the *C. albicans sur7*Δ null mutant is markedly abnormal.

### The *Candida albicans sur7*Δ mutant is impaired in lipase secretion but overproduces Sap2p

**Figure 8:**
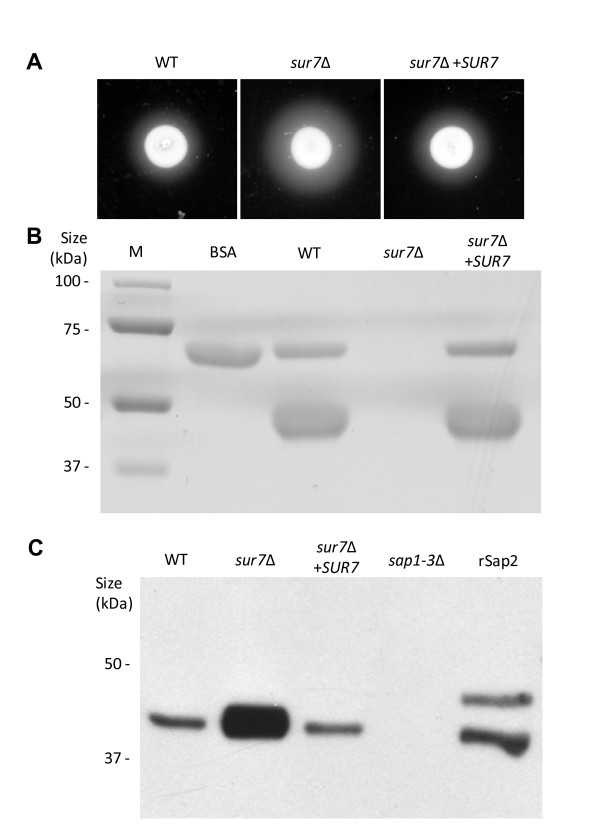
**Protease secretion in the *sur7*Δ null mutant strain**. (**A**) Extracellular protease secretion was assayed using a BSA degradation plate assay. Overnight cultures were spotted onto BSA plates and incubated at 30°C for 24 and 48 h. The relative amount of extracellular protease activity is indicated by the halo surrounding the fungal colony. (**B**) BSA degradation and Sap2p levels in liquid cultures were also assessed. Overnight cultures were shifted to medium containing BSA as the sole nitrogen source and incubated at 30°C for 6 hours. The degree of proteolysis of BSA was analyzed by reducing SDS-PAGE and Coomassie blue staining. Samples were normalized for loading with respect to culture density. Lanes containing standard protein markers (M) and intact BSA are shown for reference. (**C**) Cell-free supernatants prepared as described in (B) were analyzed by Western blotting using anti-Sap2p antibodies (from M. Monod). The triple deletion mutant strain *sap1-3*Δ (from B. Hube) was used as a negative control. rSap2 indicates purified recombinant Sap2p (from M. Monod) used as a positive control.

Secretion of degradative enzymes is important to pathogenesis, thus we characterized secretion of aspartyl proteases (Saps), lipase, and phospholipases in the *sur7*Δ null mutant strain. When inoculated on medium containing BSA as the sole nitrogen source, wild-type *C. albicans *secretes aspartyl proteinases which result in a halo surrounding the colony due to extracellular proteolysis. Compared to prototrophic control strain DAY185 and the isogenic complemented strain, the *C. albicans sur7*Δ null mutant secreted increased extracellular proteolytic activity on BSA plates (Fig. 8A) and in liquid BSA (Fig. 8B). We next examined extracellular Sap2p secretion using Western blotting of culture supernatants using anti-Sap2p antibodies (from M. Monod). The *C. albicans sur7*Δ mutant produced substantially greater amounts of extracellular Sap2p, compared to DAY185 and the complemented strain (Fig. 8C). Thus, the proteolytic degradation of BSA (Fig. 8A and 8B) is likely due to increased secretion of Sap2p. In contrast, the *C. albicans sur7*Δ mutant secreted slightly reduced amounts of extracellular Sap4-6p compared to the control and complemented strain when analyzed by Western blotting (data not shown).

**Figure 9:**
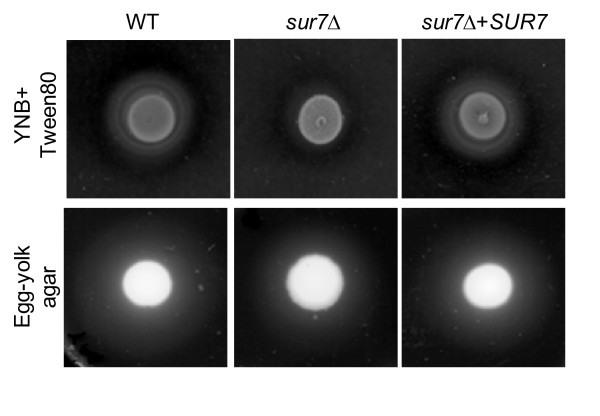
**Extracellular lipolytic activity of the *C. albicans sur7*Δ null mutant**. Overnight cultures were spotted onto YNB-Tween 80 and Egg-yolk agar plates and incubated at 37°C. The relative amount of lipolytic and phospholytic degradation is indicated by the halo of precipitation surrounding the fungal colony. Phospholipases and lipases are active on Egg-yolk agar medium whereas only lipases are active on YNB-Tween 80 agar medium [28].

We next assayed secreted phospholipase and lipase activity on egg-yolk agar and YNB-Tween 80 plates, respectively. Both phospholipase and lipase are active on egg-yolk agar plates whereas YNB-Tween 80 plates are more specific for lipase activity [28]. The *sur7*Δ null mutant strain produced almost undetectable amounts of precipitation on YNB-Tween plates but only slightly less degradative activity on egg-yolk agar plates compared with control strain DAY185 and the *SUR7 *complemented strain (Fig. 9), thus suggesting impaired lipase secretion.

### Absence of *C. albicans SUR7 *increases adherence

Adhesion plays a critical role in the early stages of *C. albicans *infection, and several secreted and cell wall-associated proteins contribute to this mechanism of pathogenesis (reviewed in [29] and [30]). Thus, given the observed secretory and cell wall defects of the *sur7*Δ strain, we next compared the degree of adhesion between the *sur7*Δ null mutant and control strains using a standard assay for adherence to polystyrene. Adherence was assayed in both RPMI-1640 (filamentation-inducing conditions) and PBS (non-inducing conditions). An increase in adherence was observed in the *sur7*Δ null mutant strain compared to *SUR7*^+ ^strains (Table 3, p < 0.0001) in either RPMI-1640 or PBS.

**Table 3:** Adhesion of *C. albicans *strains to polystyrene.

	Relative adherence units		
	**WT**	*sur7*Δ*	***sur7*Δ +*SUR7***

**PBS**	0.798 ± 0.024	1.310 ± 0.035	0.801 ± 0.012

**RPMI-1640**	0.621 ± 0.006	0.776 ± 0.007	0.643 0.019

*p-value < 0.0001

### The *C. albicans sur7*Δ mutant forms an aberrant biofilm

**Figure 10:**
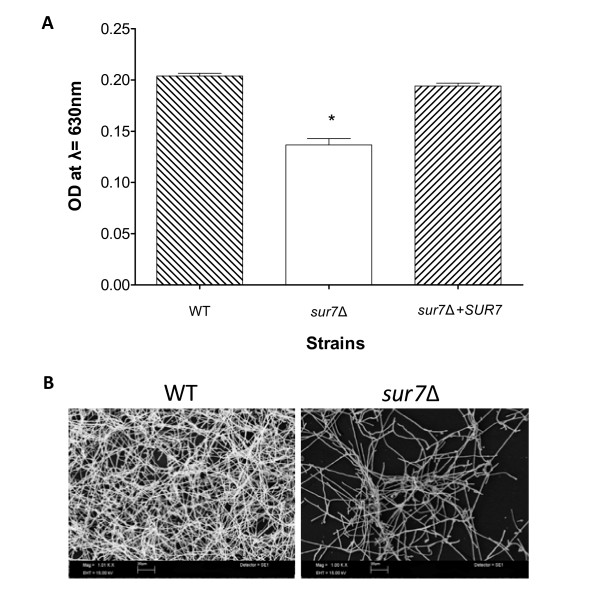
**Analysis of *C. albicans sur7*Δ biofilm formation**. (**A**) Biofilm mass was assayed by staining the biofilm formed with Crystal Violet [45]. Data analyzed consisted of 14 replicates and statistical significance was determined by ANOVA (p-value < 0.0001), indicated on the figure with an asterisk (*). (**B**) The structure and morphology of the biofilm formed by the *sur7*Δ null mutant strain and wild-type strain DAY185 was examined by scanning electron microscopy. Size bars indicate 20 μm.

We next examined the role of *C. albicans SUR7 *in biofilm formation, a key contributor to *Candida *pathogenesis. The *C. albicans sur7*Δ mutant formed a sparse biofilm, with a patchy distribution when examined by light microscopy (data not shown). Because the *C. albicans sur7*Δ mutant in planktonic culture generated increased XTT activity compared to controls (data not shown), we used an alternative method to measure biofilm mass. Analysis of biofilm mass using crystal violet staining showed a decrease in biofilm mass consistent with direct microscopic observation of the biofilm formed (Fig. 10A). Average OD_630 nm _measurements of the crystal violet extracts, which are directly related to biofilm mass, were 0.204 ± 0.003, 0.137 ± 0.006, and 0.194 ± 0.003 for the wild-type, *sur7*Δ null mutant, and *SUR7 *complemented strains, respectively (p < 0.0001). Examination of the biofilm by scanning electron microscopy demonstrated a sparse biofilm architecture compared to control strain DAY185 (Fig. 10B).

Next, in order to determine if the reduced biofilm mass of the *sur7*Δ mutant is related to decreased attachment of the biofilm, we quantified the amount of planktonic cells in the biofilm wash of each strain. Compared to the control strains, there were significantly fewer planktonic cells (colony forming units) present in the biofilm formed by the *sur7*Δ null mutant (p < 0.0001; data not shown). These results are therefore consistent with the previous adhesion studies. Thus, reduced attachment of cells does not account for the lesser biofilm mass of the *sur7*Δ null mutant. Furthermore, as there is only a minor delay or impairment in filamentation in this growth medium, it appears that the defect in biofilm formation is most likely due to a defect in cell wall or plasma membrane structure related to the absence of *SUR7*.

### The *C. albicans sur7*Δ mutant is defective in macrophage killing

**Figure 11:**
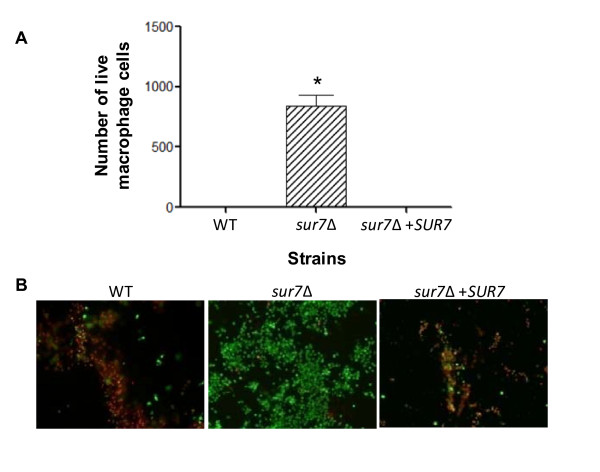
***In vitro *test of virulence using the macrophage killing assay**. Macrophages were seeded onto a glass slide and subsequently co-incubated with *C. albicans *strains at a multiplicity of infection of 2. (**A**) Live macrophage cells from four fields per strain tested were counted and the averages were compared using ANOVA. Results from co-incubations at T = 24 hours are shown. An asterisk (*) indicates statistical significance, p < 0.0001. (**B**) Live (green) and dead (red) macrophage cells were co-stained with calcein AM and ethidium bromide homodimer-1 (LIVE/DEAD Viability/Cytotoxicity kit, Invitrogen), respectively, and visualized by fluorescence microscopy. Representative images from the 24 hour timepoint for each strain are shown.

Lastly, we sought to determine the effect of the loss-of-function of *SUR7 *on the ability of *C*. *albicans *to kill macrophage cells. At early time points (1 and 5 hours co-incubation), the number of live macrophage cells co-incubated with the *sur7*Δ null mutant was similar to the numbers found when co-incubated with either DAY185 or the *SUR7 *complemented strain (>1,000 macrophages per field; data not shown). After 24 hours of co-incubation, significantly more macrophages per field remained when co-incubated with the *sur7*Δ null mutant (841 ± 87) than either of the control strains (5 ± 2 and 3 ± 1 for wild-type and *SUR7 *complemented strains, respectively) (Fig. 11A and 11B p < 0.0001). These results indicate that *C. albicans SUR7 *is required for *in vitro *macrophage killing.

## Discussion

Using a bioinformatics approach, we previously identified predicted secretion pathway proteins in *Candida albicans *[14] and next compared this with published transcriptional profiling data to identify genes highly expressed during conditions similar to bloodstream infection [15]. This approach identified a number of genes known to be involved in pathogenesis, among them *SUN41 *and *SOD5*, which have recently been studied in detail [17-21]. Among several other unknown open reading frames, we identified the *C. albicans *homolog of *S. cerevisiae SUR7*, which has recently been described in *C. albicans *as required for proper plasma membrane organization and cell wall synthesis [2]. Thus, we sought to investigate the role of *C. albicans SUR7 *in virulence-related phenotypes, including filamentation, aspartyl protease (Sap) and lipase secretion, biofilm formation, and virulence using an *in vitro *macrophage killing model.

We first assessed the structural role of *C. albicans SUR7 *from a general cellular and physiologic perspective. Loss-of-function of *SUR7 *resulted in the formation of aberrant plasma membrane invaginations and accumulation of subcellular structures inside the *C. albicans *cells, whether in the hyphal or the yeast form. Similar invaginations were observed in a *S. cerevisiae pil1*Δ deletion mutant [3], and *S. cerevisiae *Pil1p has been shown to be involved in the localization of *S. cerevisiae *Sur7p to the plasma membrane. In addition, the *C. albicans sur7*Δ mutant was hyper-susceptible to sub-inhibitory concentrations of caspofungin but not to either amphotericin B or 5-fluorocytosine. Caspofungin inhibits β-1,3-glucan synthase thus altering cell wall composition leading to cell lysis of *Candida *cells [31]. Moreover, we have demonstrated that growth of the *sur7*Δ null mutant was sensitive to SDS, Congo Red, and Calcofluor White. These results suggest that *SUR7 *plays a role in maintenance of cell wall integrity of both the yeast and filamentous form of *C. albicans*.

There was no impairment in the ability of the *sur7*Δ null mutant strain to tolerate general osmotic stresses or growth at 37°C. Likewise, in *S. cerevisiae*, the growth of the *sur7*Δ mutant, and null mutants of the *SUR7 *paralogs *ynl194*Δ and *ydl222*Δ strains was similar to wild-type under conditions of high salt or elevated temperatures [4]. However, growth of the *C. albicans sur7*Δ mutant was markedly impaired at 42°C, a phenotype that was partially rescued by the addition of 1.0 M NaCl. We demonstrated that the fluorescently-tagged *C. albicans *Sur7p paralog Fmp45p co-localizes with Sur7p-GFP. We further demonstrated increased fluorescence intensity of the *C. albicans *Sur7p paralog Fmp45p, in the presence of high salt (1.0 M NaCl) in both the *SUR7*^+ ^and *SUR7*^- ^strains. Thus the cellular localization and increased fluorescence intensities suggest that Fmp45p may play a role in survival at high temperature and salt conditions in the *sur7*Δ mutant. This suggests functional similarities between *SUR7 *and *FMP45 *that are important for growth and survival in more extreme environmental conditions. We have so far been unsuccessful in our efforts to generate a *C. albicans sur7*Δ *fmp45*Δ null mutant, and it remains to be determined if these genes are synthetic lethal in *C. albicans*.

There is limited data on the role of endocytosis in *Candida *pathogenesis. Previously, *C. albicans *ORFs homologous to *S. cerevisiae *endocytosis genes were investigated for their involvement in polarized cell growth [32]. Specifically, the authors examined *ABP1*, *BZZ1*, *EDE1*, and *PAN1*, whose gene products are involved in the early stages of endocytosis [33]. Loss of function of *PAN1*, but not *ABP1*, *BZZ1*, or *EDE1*, resulted in altered hyphal formation [32]. More recently, Douglas et al [34] investigated the role of *C. albicans RVS161 *and *RVS167 *whose homologues in *S. cerevisiae *are involved in the severance of budding endocytic vesicles from the plasma membrane. Deletion of these genes resulted in strains that produced aberrant filamentous structures and exhibited decreased virulence in a mouse model of disseminated candidiasis [34]. In *S. cerevisiae, SUR7 *localizes to eisosomes which are immobile protein assemblies that mark sites on the plasma membrane for endocytosis [3]. Defective endocytosis as a result of the deletion of *SUR7 *in *C. albicans *has been described for the yeast form of this important pathogen [2]. However, the role of *C. albicans SUR7 *in pathogenesis has not been previously examined. We present here results of experiments whose main focus was to characterize the structural and physiologic role of *C. albicans SUR7*, in order to provide a foundation to understanding the role of *SUR7 *in pathogenesis.

Thus, we next turned our attention to assessing the functional contribution of *C. albicans SUR7 *to several key virulence-related attributes. The *C. albicans sur7*Δ mutant was delayed in filamentation when induced on solid media, although this overall defect was minor. Microscopic examination revealed that the *sur7*Δ filaments branched extensively, and ultrastructurally contained subcellular structures resembling those seen in the *C. albicans sur7*Δ yeast cells. Alvarez et al. [2] also describe pseudohyphal growth of the *sur7*Δ mutant strain including an apparent defect in cell polarization, as evidenced by weak filipin staining. However, it is not clear why *C. albicans SUR7 *affects Sap or lipase secretion, as there is currently little known of the role of endocytosis in the secretion of Saps, lipases, and phospholipases.

Importantly, the *C. albicans sur7*Δ mutant formed a patchy biofilm, with many areas of scant cells and filaments. As we demonstrated an increase in adhesion in the *sur7*Δ mutant, and only a minor delay in filamentation, this markedly defective biofilm cannot be attributed to reduced adhesion or defective filamentation. Instead, we postulate that the marked plasma membrane and cell wall defects that we demonstrated in the structural studies of the *sur7*Δ mutant may be responsible for this defective biofilm. Biofilm formation is a complex, still incompletely understood process. However, cell-cell communication and adhesion are an important part of biofilm formation. We suspect that the marked derangement in plasma membrane and cell wall organization may affect the ability of the *C. albicans sur7*Δ mutant to form a normal biofilm.

Alternatively, it is possible that *SUR7 *is involved in biofilm detachment, as a negative regulator. Recently, Sellam et al. [35], performed transcriptional profiling to identify genes potentially involved in biofilm detachment (where cells from a mature biofilm detach in order to spread to distant sites within the bloodstream of an infected host). In their experiments, levels of *SUR7 *transcript were down-regulated during the initial steps of biofilm detachment. During biofilm detachment, the biofilm was observed to detach from the surface in patches. This is in agreement with the patchy morphology of the biofilm formed by the *sur7*Δ homozygous null mutant strain. Thus, we present another hypothesis that *SUR7 *may be a negative regulator of biofilm detachment, and we are currently investigating the role of *SUR7 *in biofilm detachment.

We next assayed virulence in a macrophage killing assay *in vitro*. We clearly demonstrated that the *sur7*Δ mutant strain was greatly reduced in its ability to kill murine macrophage cells at 24 hours, which is similar to the virulence defect seen in a *C. albicans vps11*Δ mutant [36]. Again, we suspect that the marked abnormalities in plasma membrane and cell wall structure render the *C. albicans sur7*Δ mutant more susceptible to macrophage killing.

## Conclusions

*C. albicans SUR7 *shares some functional homology to *S. cerevisiae SUR7*, but unlike in *S. cerevisiae*, *C. albicans SUR7 *may play a role in endocytosis and the maintenance of cell wall integrity. *C. albicans SUR7 *contributes to several key virulence-related phenotypes, and thus, may have additional molecular functions in this highly adaptable, pathogenic organism. Of note, *SUR7 *appears to be fungal-specific, with no clear human homologue. Given the phenotypes we describe here and its increased expression during infection [15], we are further investigating whether *C. albicans SUR7 *plays a role in biofilm detachment and the dissemination of infection.

## Methods

### Strains and media

*C. albicans *strains used in this study are indicated in Table 1. Strains were routinely grown at 30 (C in YPD (1% yeast extract, 2% peptone, 2% glucose) supplemented with uridine (80 μg ml^-1^), or in complete synthetic medium (0.67% yeast nitrogen base without amino acids [YNB], 2% glucose, 0.079% Complete Synthetic Mixture). Filamentation was assayed at 37°C in the following media with agar: Medium 199 containing Earle's salts (Invitrogen) supplemented with L-glutamine and buffered with 150 mM HEPES to pH 7.5; RPMI-1640 supplemented with L-glutamine (US Biological) and buffered with 165 mM MOPS to pH 7.0 (referred to as "RPMI-1640" from this point onward); 10% (v/v) fetal calf serum in YPD; and Spider medium as described by Liu et al [37]. Liquid hyphal-inducing media were inoculated with cells from overnight cultures to achieve a starting density of 5 × 10^6 ^cells ml^-1^, followed by incubation with shaking at 200 rpm at indicated time points, and visualization by microscopy. Solid media were prepared by adding 2% (w/v) agar.

### Preparation of plasmid and genomic DNA

Plasmids were expanded in *Escherichia coli *DH5α competent cells (Invitrogen) grown in LB medium with ampicillin (100 μg ml^-1^) at 37°C. Plasmid DNA was prepared from *E. coli *strains using the Fast Plasmid Mini Kit™ (5PRIME) following the manufacturer's instructions. Genomic DNA was extracted from yeast cells using the Masterpure™ Yeast DNA Purification Kit (Epicentre Biotechnologies) according to manufacturer's instructions with the exception of an extended incubation step (1 hr on ice) performed after the addition of the MPC Protein Precipitation Reagent.

### Analysis and targeted disruption of *C. albicans SUR7*

The putative *C. albicans SUR7 *open reading frame (*orf19.3414*) was identified in a genome-wide search for proteins that compose predicted *C. albicans *secretion pathway proteins [14]. The most current annotation of this gene was verified at the Candida Genome Database http://www.candidagenome.org and CandidaDB http://genodb.pasteur.fr/cgi-bin/WebObjects/CandidaDB.

The *C. albicans sur7*Δ null mutant, in background strain BWP17, was generated by disrupting both chromosomal alleles of *C. albicans SUR7 *using a PCR-based gene disruption strategy [22,23]. PCR-generated amplicons were generated using the synthetic oligonucleotides shown in Table 4 and plasmid pDDB57 (from A.P. Mitchell, Carnegie Mellon Univ.) as the template. *C. albicans *BWP17 was transformed directly with the PCR reaction mixtures using the lithium acetate method. Uridine prototrophs were selected and purified on synthetic media lacking uracil and uridine, genomic DNA was extracted using the Masterpure™ Yeast DNA Kit (Epicentre), and homologous integration of the gene targeting cassette was verified by allele-specific PCR, using one primer upstream and one primer downstream of the open reading frame and outside of the targeting region of the disruption cassette (Table 4).

**Table 4:** Primer sequences used in this study.

Primer	Primer sequence (5' → 3')
IPF12442-5DR	TTATTCTTTGAATTACTTTCCAACTTACTTCATTCCCAATTGATTAAAGTTATAAAGAATTAAACGAAACGTTTTCCCAGTCACGACGTT

IPF12442-3DR	TACGATTTCTTAATCAAATATCAAACTTTAACCCTCTCAAAAAGCTAATAAACTAACATTGATTACTCAATGTGGAATTGTGAGCGGATA

IPF12442-5Det	GCTATTCCACTTCCACTTGG

IPF12442-3Det	CAAGGGGGGAGAAGAATGGG

SalI-5'-IPF12442	GTTCTGTCGACTTGGTTGGTTGGTTGGTT

NdeI-3'IPF12442	CAATCCATATGTAACTCATGTCACGC

FMP45-5FP	GGCGAAAGACTCTCTAATGCATCAAAATTCAGATTTTTTAGAGTGAAAAGAGCCAAATCAGAAGATGTTGGTGGTGGTTCTAAAGGTGAAGAATTATT

FMP45-3HisR2	AAATTCATAATGGGTGTAGTTTAAGCCCAAAAAATTCAACAGTACAATCAATGAGGACTTTTTCTTCATTGAATTCCGGAATATTTATGAGAAAC

FMP45FP-5Det	CCCTCGTGGTGAATACGAATC

SUR7-5FP	GAAGAAAACACAGGCGGTATTAGATTCTTCAAAATCAAAAGAAACCAAAAAGTTTCCGATGATGAATCAGTAGGTGGTGGTTCTAAAGGTGAAGAATTATT

SUR7-3HisR2	TACGATTTCTTAATCAAATATCAAACTTTAACCCTCTCAAAAAGCTAATAAACTAACATTGATTACTCAAGAATTCCGGAATATTTATGAGAAAC

SUR7FP-5Det	CTAGAGCTGCCCCACCAACT

FMP45-3UraR1	AAATTCATAATGGGTGTAGTTTAAGCCCAAAAAATTCAACAGTACAATCAATGAGGACTTTTTCTTCATTTCTAGAAGGACCACCTTTGATTG

ADHTERAS [39]	GAGATATCGATCCTAGCGTAG

3FP-URADet	GTGACACCATGAGCATTGGT

To disrupt the second *SUR7 *allele, selected *C. albicans SUR7*/*sur7Δ::dpl200-URA3-dpl200 *mutants were transformed with the PCR-generated gene disruption cassette, similar to the process of creating the first allele knockout strains, except plasmid pRS-Arg4ΔSpeI [22] was used as the template. Histidine prototrophy was restored after transforming the resulting strain with *Nru*I-linearized pGEM-HIS1 [22].

In order to generate an isogenic *SUR7 *complemented strain, a copy of wild-type *SUR7 *was sub-cloned into pGEM-HIS1, digested with *Nru*I, and transformed into the *sur7Δ*::*URA3/sur7Δ*::*ARG4 *strain. Reverse and forward sequencing of the cloned *SUR7 *gene was performed, and confirmed that the sequence was identical to the CGD Assembly 21 *SUR7 *sequence. Correct integration of the wild-type gene was confirmed by allele-specific PCR in multiple independent transformants. Standard methods were used for restriction mapping, subcloning, DNA sequencing, and lithium acetate transformation [38].

Strain construction was verified by Southern blotting and standard blotting and hybridization techniques [38]. Briefly, genomic DNA digested with *Hin*d III and *Cla *I, was run on a 0.8% (w/v) agarose gel. DNA fragments were subsequently transferred by capillary action to a positively charged nylon membrane (Roche Applied Science) using 20× Saline Sodium Citrate buffer. A 1.1 kb DIG-labelled PCR amplicon from *C. albicans SUR7 *(n.t. -585 to +541 of *orf19.3414*) was then used to probe the membrane. Detection of *Hin*d III/*Cla *I DNA fragments of the expected band sizes for the wild-type allele (*SUR7*; 3.6 kb), first (*sur7*Δ::*URA3*; 2.5 kb) and second (*sur7*Δ::*ARG4*; 1.4 kb) allele knockout cassettes confirmed the genotype of each strain used in this study (Additional File 1).

### Construction and analysis of *FMP45*-GFP tagged *C. albicans *strains

Green-fluorescent protein-tagged (GFP) strains of *C. albicans FMP45 *(*orf19.6489*) were generated using PCR-mediated insertion of GFP according to published methods, using primers FMP45-5FP and FMP45-3HisR2 and plasmid pMG1646 (pGFP-HIS1) as a template [39]. This method allows for genomic integration of GFP-coding sequences at the C-terminus of the protein of interest while maintaining control of expression of the fusion protein under its native promoter. Transformants were selected on medium lacking histidine, and confirmation of correct integration into strains BWP17 (*SUR7*/*SUR7*) and SMB3 (*sur7*Δ/*sur7*Δ) was performed by allele-specific PCR on genomic DNA extracted from independent transformants. Localization of Fmp45p-GFP was performed using laser scanning confocal microscopy of live cells grown in complete synthetic medium in the presence or absence of 1.0 M NaCl at 42°C. Images were acquired on the Zeiss LSM700 on an Axio Observer Z1 (Carl Zeiss MicroImaging Inc). Image J software (National Institutes of Health; http://rsb.info.nih.gov/ij) was used to quantify fluorescence intensity of representative cells using the Plot Profile function. Median fluorescence intensity indicates the overall fluorescence intensity of a representative cell.

Additionally, a double fluorescent tagged strain was constructed to study the cellular localization of Fmp45p with respect to Sur7p localization. First we created a *SUR7*-YFP strain as described in the previous paragraph except that the PCR amplicon used was generated using pMG1656 (pYFP-HIS) [39] and primers SUR7-5FP and SUR7-3HisR2 (Table 4). The resulting strain was next transformed with PCR amplicons generated using primers FMP45-5FP and FMP45-3UraR1 and pMG1602 (pGFP-URA) [39] and transformants were selected on medium lacking uracil and uridine. An additional control strain, *SUR7*-GFP, was also created using pMG1646 (pGFP-HIS) as a template [39] and primers SUR7-5FP and SUR7-3HisR2. Correct integration of the *SUR7*-*YFP*, *SUR7*-*GFP*, and *FMP45*-*GFP *alleles were verified by allele-specific PCR on genomic DNA extracted from independent transformants, using primer pairs SUR7FP-5Det and ADHTERAS; and FMP45FP-5Det and 3FP-URADet, respectively. Images were acquired on a Zeiss Axioskop 2MOT microscope using the Nuance™ Multispectral Imaging System (CRi). Using the microscope's green fluorescence filter set (Ex: 475/28 nm; Em: 515 nm LP; Single-band dichroic: 519 nm), a series of images (spectral cube) was acquired at 10 nm intervals from 500 - 720 nm as defined by the Nuance™ system's liquid crystal tunable filter. Spectral cube images were acquired from control strains: auto-fluorescence (DAY185), YFP only (*SUR7*-YFP), and GFP only (*SUR7*-GFP), as well as from the *SUR7*-YFP *FMP45*-GFP multiply-expressing strain. Using Nuance software, pure spectra were generated for autofluorescence, GFP and YFP which were subsequently used to unmix spectral cubes acquired of the *SUR7*-YFP *FMP45*-GFP strain. Following linear unmixing, the individual fluorophore-tagged proteins were viewed in separate component images, with the extent of GFP-YFP co-localization indicated in a merged image. Although there is significant overlap of the fluorescence emission spectra of GFP and YFP that cannot be distinguished using conventional epifluorescence imaging, acquisition and unmixing of spectral images using the Nuance™ spectral imaging camera allowed these spectrally distinct fluorophores to be individually detected. Details of strains and primers used in this study are found in Tables 1 and 4.

### Analysis of growth and stress tolerance

Growth was assessed in liquid media by measuring OD_600 _at fixed intervals in an automated Bioscreen C Analyzer (Thermo Labsystems) as described previously [40]. Growth curves were generated using Prism 5.0 (GraphPad Software, Inc). Strains were analyzed in conditions of high osmolar stress (2.5 M glycerol or 1.0 M NaCl) and varying temperatures (30, 37, or 42°C). Strains grown in complete synthetic medium supplemented with uridine were also exposed to sub-inhibitory concentrations of caspofungin, amphotericin B, and 5-fluorocytosine. Pilot growth curves were first generated to determine the working concentration of each antifungal reagent to be used. The final concentrations of caspofungin, amphotericin B, and 5-fluorocytosine used for phenotypic analysis were 0.25, 0.5, and 10 μg ml^-1^, respectively. Cell wall integrity was tested on YPD agar medium containing Calcofluor White (20, 50, and 125 μg ml^-1^), caspofungin (0.1, 0.2, and 0.5 μg ml^-1^), Congo Red (50, 100, and 200 μg ml^-1^), or SDS (0.01%, 0.02%, and 0.05%).

### Endocytosis assay and visualization of vacuolar morphology

The effect of the loss of function of *SUR7 *on endocytosis in both the yeast and filamentous forms was assessed by following the fate of the lypophilic dye FM4-64 [41]. For dual staining with carboxy-DCFDA (CDCFDA; Invitrogen), cells were first stained with FM4-64. Next, 1.0 × 10^6 ^cells ml^-1 ^were resuspended in 50 mM sodium citrate buffer and CDCFDA was added according to the manufacturer's instructions. Following a 15 minute incubation period at room temperature, microscopy then was performed on a Nikon Eclipse 80i fluorescent microscope (Nikon Instruments, Inc.). Images were acquired and color added using NIS-Elements Documentation software, version 2.34 (Nikon Instruments, Inc.).

### Enzyme assays

Extracellular protease secretion was assayed on BSA plates [42]. Lipase activity was visualized on egg-yolk agar plates and YNB agar containing 2.5% (v/v) Tween 80 [28]. BSA degradation in liquid media was assessed by SDS-PAGE, followed by analysis of Sap2p secretion by Western blot analysis as described [43], except that the medium used was 1.18% Yeast Carbon Base (YCB, Difco™), 0.01% yeast extract, and 0.1% BSA. Equivolumes of culture supernatants were loaded from each strain, using the same concentration of cells for each strain. Saps 4-6p were induced by growing strains, at a starting concentration of 5 × 10^6 ^cells ml^-1 ^in RPMI-1640, at 37°C for 24 h with shaking at 200 rpm. Saps 4-6p secreted into the media were analyzed by Western blot using anti-Sap5p polyclonal antibody (from M. Monod), after transferring to PVDF membrane (Bio-Rad), and detection using chemiluminescence (ECL Plus Detection Kit, GE Healthcare Life Sciences) and autoradiography.

### Adhesion assays

Adhesion to polystyrene was assessed as described previously [44] with slight modifications. An inoculum of 1.0 × 10^7 ^cells ml^-1 ^in PBS or RPMI-1640 was prepared, of which 150 μl was added to individual wells of a 96-well microtiter plate. An identical set of each strain was dispensed into individual microcentrifuge tubes for use as the unwashed control, representing the total number of adherent and non-adherent cells. Following a 2-hr incubation period at 37°C, non-adherent cells were removed by washing the wells of the microtiter plate, while cells incubated in microcentrifuge tubes were pelleted at high speed on a benchtop centrifuge. The XTT-reduction assay [45] was then used to quantify the adhered and total amount of cells in each well and microcentrifuge tube, respectively. After incubation with the XTT-menadione substrate at 37°C for 3 hours, 75 μl of colored formazan was transferred to a fresh microtiter plate and absorbance was read at 490 nm. The adherence capacity of each strain was calculated as the mean XTT-value of the washed cells relative to the mean XTT-value of the unwashed cells. Experiments were performed at least twice, each with 8 replicates per strain tested. Statistical significance was assessed with an analysis of variance (ANOVA) between all strains compared using Prism 5.0 (GraphPad Software, Inc.)

### Analysis of fungal biofilms

Formation of *C. albicans *biofilms and the XTT-reduction assay were performed as previously described [45]. As an alternative, Crystal Violet staining was used to estimate the biofilm mass [45]. Briefly, biofilms were stained with 100 μl of a 0.3% (w/v) Crystal Violet, 5% (v/v) isopropanol, 5% (v/v) methanol solution for 5 min, after which the wells were washed with water. The dye was then extracted from the biofilms using 100 μl ethanol, of which 75 μl was transferred to a clean microtiter plate and the absorbance was measured spectrophotometrically at 550 nm. Scanning electron microscopy was performed on biofilm samples formed on a coverslip (Thermanox, Nalge Nunc International) after 24 h incubation of a 0.5 ml inoculum containing 1.0 × 10^6 ^cells ml^-1 ^according to previously described methods [46]. Non-adherent, planktonic cells were quantified from biofilm washes by plating serial dilutions of the pooled washes, from individual replicates, onto YPD agar plates, and colony forming units were determined following incubation at 30°C for 2 days.

### Macrophage killing assays

The macrophage killing assay was performed as described by Palmer et al. [36]. The murine macrophage cell line used in this study, J774A.1, was purchased from ATCC and propagated in high glucose D-MEM supplemented with 10% FCS. Next, 2.0 × 10^5 ^J774A.1 cells in a volume of 0.75 ml were seeded in Lab-Tek Chambered Slides (Nalge-Nunc), incubated overnight at 37°C with 5% CO_2_. *C. albicans *strains, diluted and grown as previously described [47], were co-incubated with the adhered macrophage at a multiplicity of infection (MOI) of 2, for a specified period of time. Following co-incubation, the cells were washed twice with phosphate-buffered saline (PBS) and viability was assessed using 0.2 μM calcein AM and 4 μM ethidium bromide homodimer (LIVE/DEAD Viability/Cytotoxicity kit, Invitrogen) according to the manufacturer's instructions. Live macrophages from four fields of each chamber were counted and statistical differences between the average values was assessed using ANOVA followed by Tukey's multiple comparison of means.

## Authors' contributions

SMB participated in the design and performed all experimentation presented in the manuscript, except where acknowledged in appropriate section(s). SAL conceived of the study, correlated the bioinformatics data with published *in vivo *and *in vitro *transcription profiling data, and participated in the design of the experiments. All authors contributed towards the analysis and interpretation of results towards intellectually significant findings, drafted, read, and approved the final manuscript for submission.

## Authors' information

SAL is a physician-scientist (MD, Ph.D) who is the Chief of Infectious Diseases at the New Mexico VA Healthcare System, and Assistant Professor at the School of Medicine of the University of New Mexico (Albuquerque, NM).

## Supplementary Material

Additional file 1 **Confirmation of *sur7*Δ heterozygous and homozygous null mutants by Southern blot**. Southern hybridization was performed on *Hin*d III-*Cla *I digests of genomic DNA of transformants of interest using a DIG-labeled probe that hybridizes to n.t. -585 to +541 of *C. albicans SUR7*. The expected sizes of the restriction fragments are: wild-type (*SUR7*) allele 3.6 kb, 1^st ^allele gene replacement (*sur7*Δ::*URA3*) 2.5 kb, and 2^nd ^allele gene replacement (*sur7*Δ::*ARG4*) 1.4 kb. Genomic DNA from the wild-type strain (*SUR7/SUR7*), DAY185, was run in the first lane marked "WT". Genomic DNA from a heterozygous null mutant (*sur7*Δ/*SUR7*) isolate was run in the second lane marked "Δ/+". Genomic DNA from two independent homozygous null mutant strains (*sur7*Δ/*sur7*Δ) was run in the lanes marked "Δ/Δ". Size markers from standard *Hin*d III digest of lambda DNA is shown on the left for reference. Click here for file
