# Supplementary figures and images for: Candida albicans SUR7 contributes to secretion, biofilm formation, and macrophage killing
Source: BMC Microbiol. 2010 Apr 30;10:133. doi: 10.1186/1471-2180-10-133 (PMC2887802; doi:10.1186/1471-2180-10-133)

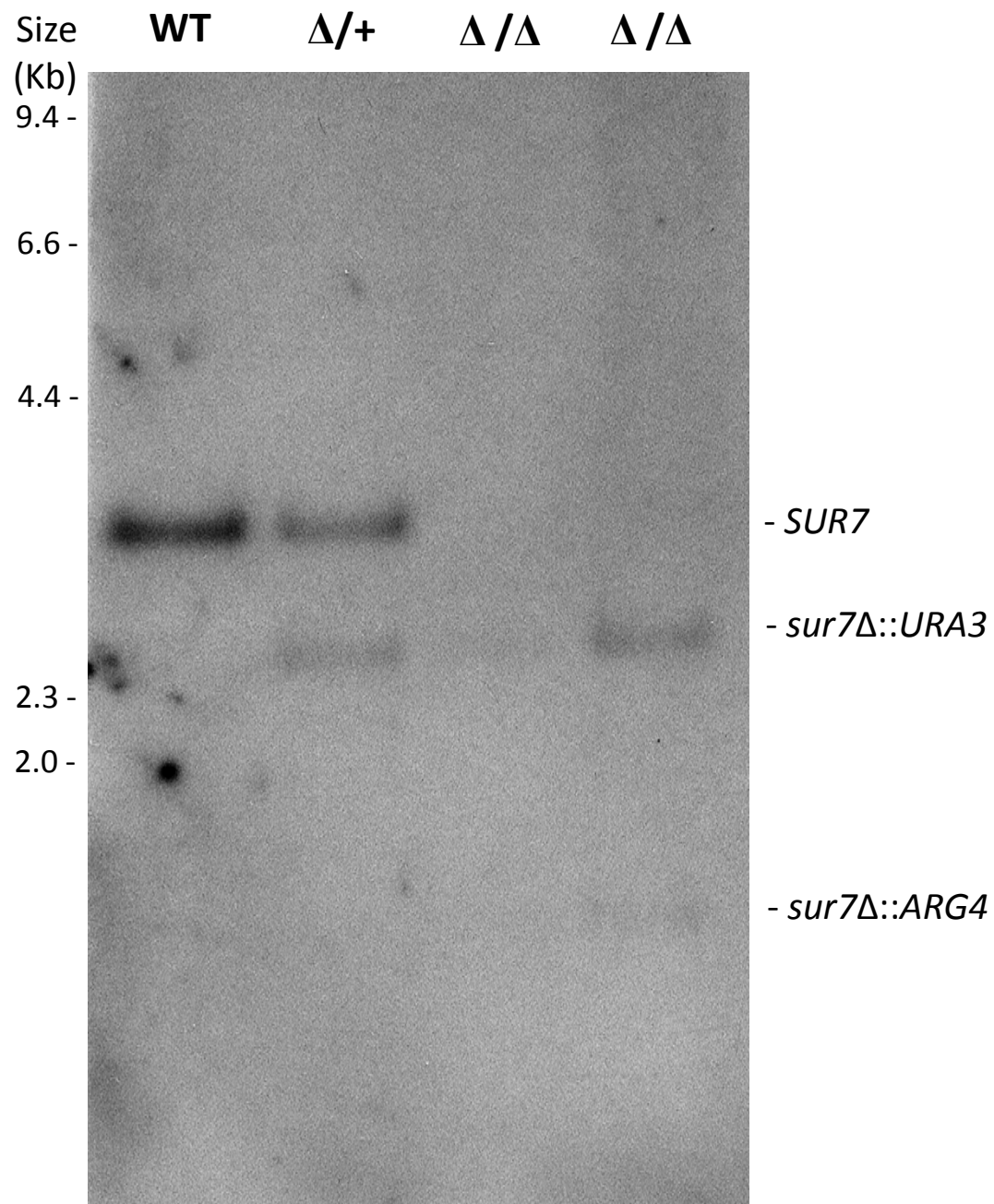

Supplement: Additional file 1 — Confirmation of sur7Δ heterozygous and homozygous null mutants by Southern blot. Southern hybridization was performed on Hind III-Cla I digests of genomic DNA of transformants of interest using a DIG-labeled probe that hybridizes to n.t. -585 to +541 of C. albicans SUR7. The expected sizes of the restriction fragments are: wild-type (SUR7) allele 3.6 kb, 1st allele gene replacement (sur7Δ::URA3) 2.5 kb, and 2nd allele gene replacement (sur7Δ::ARG4) 1.4 kb. Genomic DNA from the wild-type strain (SUR7/SUR7), DAY185, was run in the first lane marked "WT". Genomic DNA from a heterozygous null mutant (sur7Δ/SUR7) isolate was run in the second lane marked "Δ/+". Genomic DNA from two independent homozygous null mutant strains (sur7Δ/sur7Δ) was run in the lanes marked "Δ/Δ". Size markers from standard Hind III digest of lambda DNA is shown on the left for reference. [file 1471-2180-10-133-S1.PDF]
